# Optimizing Transformation Frequency of *Cryptococcus neoformans* and *Cryptococcus gattii* Using *Agrobacterium tumefaciens*

**DOI:** 10.3390/jof7070520

**Published:** 2021-06-29

**Authors:** Jianmin Fu, Nohelli E. Brockman, Brian L. Wickes

**Affiliations:** Health Science Center, Department of Microbiology, Immunology, and Molecular Genetics, University of Texas, San Antonio, TX 78229-3900, USA; fuj@uthscsa.edu (J.F.); brockmann@livemail.uthscsa.edu (N.E.B.)

**Keywords:** transformation, bacterial, Ti-plasmid

## Abstract

The transformation of *Cryptococcus* spp. by *Agrobacterium tumefaciens* has proven to be a useful genetic tool. A number of factors affect transformation frequency. These factors include acetosyringone concentration, bacterial cell to yeast cell ratio, cell wall damage, and agar concentration. Agar concentration was found to have a significant effect on the transformant number as transformants increased with agar concentration across all four serotypes. When infection time points were tested, higher agar concentrations were found to result in an earlier transfer of the Ti-plasmid to the yeast cell, with the earliest transformant appearing two h after *A. tumefaciens* contact with yeast cells. These results demonstrate that *A. tumefaciens* transformation efficiency can be affected by a variety of factors and continued investigation of these factors can lead to improvements in specific *A. tumefaciens*/fungus transformation systems.

## 1. Introduction

While model fungi have yielded incredible insight into molecular biology and genetics, due in large part to their ease of manipulation, human fungal pathogens are much more difficult to work with. A few have easily maintained episomal plasmids, some are diploid, which until CRISPR made gene disruption laborious. Others can be hazardous to work with in the laboratory, and a number of the filamentous fungi are multinucleate. Furthermore, for most human fungal pathogens, transformation efficiencies are low compared to *Saccharomyces cerevisiae*. However, these issues have been continually addressed and have led to constant improvements in the molecular toolboxes of pathogenic fungi.

One tool that has been widely applied to human fungal pathogens is transformation using *Agrobacterium tumefaciens,* a transkingdom bacterial pathogen. To date, more than 100 fungi across all phyla have been transformed using *A. tumefaciens* [1]. More importantly, *A. tumefaciens* has been used to transform both yeasts and molds [1] and has been successfully utilized for almost all of the major human fungal pathogens including *Cryptococcus* spp., *Coccidioides* spp., *Aspergillus* spp., *Histoplasma capsulatum*, *Paracoccidioides brasiliensis*, and *Blastomyces dermatitidis* [2,3,4,5,6,7,8]. The continued development of *A. tumefaciens* fungal systems has led to new and novel applications [9,10,11].

*A. tumefaciens* was initially identified as the causative agent of crown gall disease, a plant infection characterized by tumorous growth [12]. The tumor-inducing property of *A. tumefaciens* is derived from a plasmid (Ti-plasmid) containing numerous genes that encode proteins responsible for the transfer of a segment of plasmid DNA (T-DNA) to the host cell (reviewed in [13]). The transfer of T-DNA occurs through a type IV secretion system similar to bacterial conjugation [14]. After the transfer of T-DNA to the host cell, the segment typically integrates as a single copy, randomly throughout the genome. Integrated T-DNA in turn directs the host cell synthetic machinery to produce bacterial proteins responsible for tumorigenesis [15]. A key aspect of recombinant Ti-plasmids is the insertion of a suitable host-cell-specific selectable marker into the genome, which allows for the selection of T-DNA transformants after infection of the target host. Isolation and investigation of the Ti-plasmid has enabled the construction of numerous recombinant plasmids that are suitable for function in a wide variety of organisms [16].

A crucial component in extending the functionality of *A. tumefaciens* transformation is maximizing transformation efficiency. The general strategy for transformation with *A. tumefaciens* begins with separate broth cultures for the *A. tumefaciens* bacterial strain, which harbors the Ti-plasmid containing a host-specific selectable marker (usually a dominant drug-resistant marker). This culture, along with the fungal host strain grown in parallel, are harvested at log phase growth, adjusted for cell number, and then mixed at a specific ratio. The mixture is then captured on a membrane after filtration and the membrane is laid on co-culture agar, which is incubated for 1–3 days at 24–26 °C to facilitate the transfer of the T-DNA into the fungal host. Acetosyringone (a synthetic plant hormone) is required at some stage of the process for the stimulation of T-DNA transfer. After incubation on co-culture agar, cells are washed off the filter and then plated onto a selective medium for fungal transformants, which also contains an antibiotic that kills *A. tumefaciens* cells.

To date, there are many variables in the transformation protocol that have been shown to affect transformation efficiency. These include varying the acetosyringone concentration, *A. tumefaciens*/host cell concentration, and co-cultivation conditions, to name a few [1,8,17,18]. In an effort to improve the transformation frequency of *A. tumefaciens* in *Cryptococcus neoformans*, we investigated a number of factors to determine what effect they had on transformation frequency. From among the variables we investigated, agar concentration had the largest effect on transformation efficiency, yielding an almost 100× improvement over the control protocol. The improvement of transformation efficiency in *C. neoformans* potentially enables the application of high throughput screening methods for transformation libraries.

## 2. Materials and Methods

### 2.1. Media

YPD contained 2% dextrose, 2% peptone, 1% yeast extract, and when needed, was solidified with 2% agar. LB agar (1% tryptone, 0.5% yeast extract, 1% NaCl, and 1.5% agar) contained 100 μg/mL kanamycin and was used for growing *A. tumefaciens*. Transformation plates consisted of YPD with 200 μM Cefotaxime (Gold Biotechnology, St. Louis, MO, USA) and 60 μg/mL G418 sulfate (Corning, Inc., Oneonta, NY, USA). Unless otherwise indicated, media components were obtained from Difco, Inc. (Detroit, MI, USA). Chemicals were obtained from Sigma Aldrich, Inc. (St. Louis, MO, USA), unless otherwise indicated. Media and solutions (minimal media, induction media, co-cultivation agar) for *A. tumefaciens* transformation were prepared as previously described [8,19]. Minimal media, induction media, co-cultivation agar, Acetosyringone (100 mM prepared in DMSO), and 0.1% FeSO_4_ (filter-sterilized) were made fresh (same day) for each transformation.

### 2.2. Strains and Plasmids

WSA16 (serotype C, alias NIH 191), WSA21 (serotype D, alias JEC21), WSA86 (serotype B, alias NIH B3939), and H99 (serotype A) are *C. neoformans* strains in our lab stock. NIH strains were gifts from K.J. Kwon Chung, and H99 was a gift from John Perfect. *A. tumefaciens* strain EHA105 was used for all transformations and was a gift from KJ Kwon-Chung [8]. Plasmid pYCC716 [8] was used for all infections and was a gift from KJ Kwon-Chung.

### 2.3. A. tumefaciens Standard Transformation

The standard *A. tumefaciens* transformation was based on previously described methods with modifications [8,19], which was further modified according to the variable that was being tested. Briefly, *A. tumefaciens* strain EHA105 containing plasmid pYCC716 [8] was maintained as a −70 °C glycerol stock and revived by plating onto LB agar with 50 μg/mL kanamycin, followed by incubation at 28 °C overnight. *C. neoformans* strains were revived from −70 °C glycerol stocks and cultured on YPD agar at 30 °C overnight. After a second transfer and overnight growth on LB-kan plates, approximately 5 × 10^8^ *A. tumefaciens* cells from the agar plate were inoculated into 30 mL of minimal medium broth containing 100 μg/mL kanamycin in a 250 mL flask, which was shaken at 250 rpm at 28 °C overnight. At the same time, ~1 × 10^6^ *C. neoformans* cells were inoculated into 3.0 mL YPD broth in a 15 mL Falcon polypropylene snap cap tube (Fisher Scientific, Inc., Pittsburgh, PA), which was incubated in parallel with the *A. tumefaciens* culture (250 rpm at 28 °C overnight). After overnight growth, 15 mL of the *A. tumefaciens* culture were pelleted in a 50 mL screw cap Falcon tube (Fisher Scientific, Inc., Pittsburgh, PA, USA) and resuspended in 30 mL filter-sterilized induction media containing 200 μM Acetosyringone and 100 μg/mL kanamycin. The cells were shaken at 250 rpm for 6 h at 28 °C in the same tube with caps taped loosely in place. Concurrently, the *C. neoformans* culture was prepared by inoculating 0.4 mL of the overnight YPD culture into 9.6 mL of YPD broth in a 50 mL sterile Falcon tube with caps taped loosely in place. After 6 h, the *C. neoformans* culture was washed 2× in sterile water, suspended in 2.0 mL of induction media, and adjusted to 1 × 10^8^ cells/mL in the same medium by counting in a hemocytometer. The OD_660_ of the *A. tumefaciens* culture was then taken and adjusted to 1.0 in the induction medium (approx. 1 × 10^9^ cells/mL). One hundred microliters of *A. tumefaciens* cells were mixed with the same volume of *C. neoformans* cells (10:1 cell/cell ratio) and filtered through a 0.45 μm pore size, white, gridded, 13 mm mixed cellulose membrane (Millipore, Billerica, MA, USA) loaded into a 13 mm Swinney filter holder (Pall Inc., Port Washington, NY, USA) using a QiAvac 24 filtration apparatus (Qiagen, Inc., Valencia, CA, USA). Immediately after filtration, filters were placed upright on 60 mm × 15 mm co-cultivation agar plates (Fisher Scientific, Inc., Pittsburgh, PA, USA). Agar plates were then incubated at 26 °C for 1–3 days. At the end of the incubation period, filters were transferred to a 2.0 mL screw cap tube (Sarstedt, Numbrecht, Germany) and vortexed with 1.0 mL sterile water to dislodge cells. Fifty microliter aliquots of these cells, and dilutions, when necessary, were plated onto YPD agar containing 60 μg/mL G418 sulfate and 200 μM Cefotaxime (Gold Biotechnology, St. Louis, MO, USA). Plates were incubated at 30 °C for two days, unless otherwise indicated. Colony counts were performed for each plate, and then the average and standard deviation for each condition were determined. Experiments were performed in triplicate and analyzed by one-way ANOVA with post hoc Tukey Honestly Significant Difference (HSD) test, where needed, and using Excel with the Analysis Toolpak add-in (Microsoft, Inc., Redman, WA, USA).

### 2.4. The Effect of the A. tumefaciens:C. neoformans Ratio on Transformation Frequency

To determine the effect that the ratio of *A. tumefaciens* cells to *C. neoformans* cells had on transformation frequency, both cultures were grown using the standard protocol. Cells were then enumerated and mixed prior to filtration in the following ratios of *A. tumefaciens*:*C. neoformans*—1:1, 2.5:1, 5:1, 10:1, 25:1, and 50:1. After filtration, membranes were placed onto 1.5% co-culture agar for two days, then plated onto YPD with 200 μM Cefotaxime and 60 μg/mL G418 sulfate.

### 2.5. The Effect of Physical Damage on Transformation Frequency

In order to test the effect of cell wall integrity on transformation efficiency, we used a physical approach to damage the cell walls prior to infection. We use two bead beating methods in our laboratory to break fungal cell walls. A bead beater that rapidly agitates cells and beads, which can quickly lyse cells, or vortexing, which is performed on a standard vortexer (Vortex Genie, Fisher Scientific, Pittsburgh, PA, USA) and causes less damage. We decided to use vortexing because cell damage is slower and easier to control. Bead beating was performed by transferring 1.0 mL of induction medium containing 1 × 10^8^ cells of WSA21 into a screw cap tube and then adding 500 μL (*v*:*v*) of 1.0 mm diameter glass beads (Biospec Products, Bartlesville, OK, USA). Tubes were vortexed at the highest setting for 3, 6, 9, 12, 15, or 18 min. Cell suspensions were carefully removed and placed in a new tube, leaving beads behind, which settle to the bottom of the tube without the need for centrifugation. After bringing the volume back to 1.0 mL, these cells were then immediately transformed with *A. tumefaciens,* as above.

### 2.6. The Effect of Acetosyringone Concentration on Transformation Frequency

The effect of acetosyringone concentration on transformation frequency was tested in three different ways. Acetosyringone was added to the induction media only, in varying concentrations, while no acetosyringone was added to the co-culture medium. We also added acetosyringone only to the co-culture medium, while the induction medium amount was kept at zero. Finally, acetosyringone was added to both the induction and co-culture media at the same concentrations. For each condition, we used the same concentration series, which ranged from 0 μM (control) to 7× the concentration of the standard transformation protocol.

### 2.7. Agar Concentration Effect on Transformation Frequency

The effect of agar concentration on transformation efficiency was determined by placing membrane filters onto co-culture agar containing 1.5%, 2.0%, 3.0%, 4.0%, 6.0%, and 8.0% agar after filtration, and incubating for 1, 2, or 3 days at 30 °C, and then counted. Plates were poured on the same day of the experiment and dried in a biohazard hood to remove all visible moisture from the surface.

### 2.8. A. tumefaciens Infection Time Course

Plasmid transfer times after *A. tumefaciens* exposure to *C. neoformans* were determined by using the standard *A. tumefaciens* protocol to infect WSA21. After filtration onto the membrane filter, filters were placed onto co-culture agar solidified with either 1.5%, 4%, or 8% agar, then removed hourly at indicated times. Cells were recovered from filters and plated onto YPD with G418 sulfate and Cefotaxime.

### 2.9. Determination of A. tumefaciens Integration Sites

The percentage of *A. tumefaciens* transformants with randomly integrated or multicopy insertions was determined by screening for HinP1I (a four-base pair cutter) (New England Biolabs, Beverly, MA, USA) restriction sites in flanking host DNA using vectorette PCR [20]. DNA from 24 randomly chosen transformants was isolated, as previously described using 5 × 10^8^ cfu of yeast cells that were recovered from a patch grown for 24 h on YPD [21]. DNA was prepared for vectorette PCR by digesting 1 μg with HinP1I, according to manufacturer’s instructions, then cleaned using the QiaQuick PCR Purification kit (Qiagen, Valencia, CA, USA). Vectorette adapters prepared from Vect53 and Vect57GC [22] were ligated to digested DNA using T4 ligase (Invitrogen, Inc., Carlsbad, CA, USA), according to manufacturer’s instructions. Insertion junctions were identified by amplifying a 1:100 dilution of the ligation reaction using KOD Xtreme Hot Start Taq polymerase (Sigma Aldrich, St, Louis, MO, USA) and primers vectB21 (CGTAACCGTTCGTACGAGAAT) and pYCC.left (for left flank insertion site) (CGGCCGTTACTAGTGGATCT) or pYCC.right (for right flank insertion site) (ATCGGCGGGGGTCATAAC) using an annealing temperature of 58 °C and 40 cycles in a 25 μL reaction, according to manufacturer’s instructions. PCR reactions were checked for amplicon sizes and integration number on a 1.0% gel. Differences in multiple integration frequency were compared by Chi square.

## 3. Results

### 3.1. Bacteria to Yeast Cell Ratio Effect on Transformation Frequency

**Figure 1:**
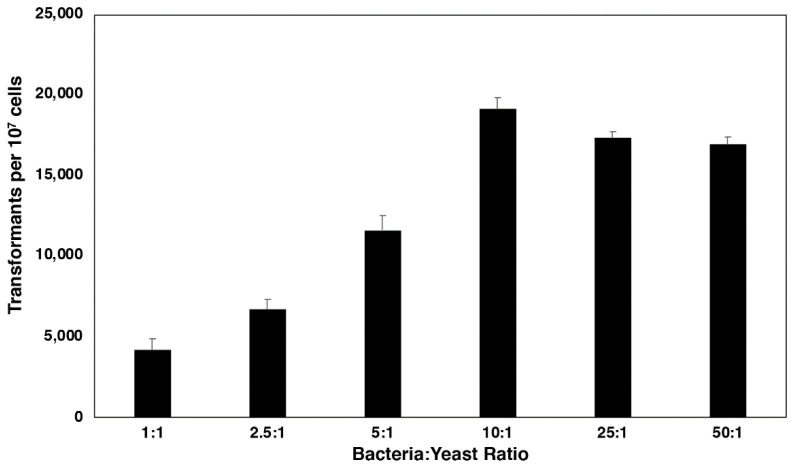
The effect of bacteria:yeast ratios on transformation frequency. *C. neoformans* and *A. tumefaciens* cells were mixed in various ratios using the standard protocol, with *C. neoformans* cells kept constant at 1 × 10^7^. Experiments were performed in triplicate with three plates per experiment and counted at 48 h. Colony counts are per 1 × 10^7^ *C. neoformans* cells. Transformant numbers were significantly different from each other for each ratio, except 25:1 and 50:1, which were not significantly different from each other (*P* < 0.05).

In an earlier study of *A. tumefaciens’* transformation of *C. neoformans*, McClelland et al. found the optimum ratio of bacteria:yeast cells to be 10:1 [8]. They found no significant difference in transformation frequency when ratios varied from 1:1 to 100:1 and concluded that 10:1 was the most efficient ratio for maximizing transformants. In our hands, we also found the highest transformation frequency at a 10:1 bacteria:yeast ratio, although at higher ratios of 25:1 or 50:1, transformant numbers slightly decreased. However, at ratios lower than 10:1, we found a significant decrease between 10:1 and 5:1 bacteria:yeast ratios, with a consistent decrease in the number of transformants as ratios decreased to 1:1. At a 1:1 ratio of bacteria to yeast cells, an almost 5-fold reduction in transformation frequency was observed compared to the maximum number of transformants recovered at a 10:1 ratio (Figure 1).

### 3.2. Physical Damage by Bead Breakage

**Figure 2:**
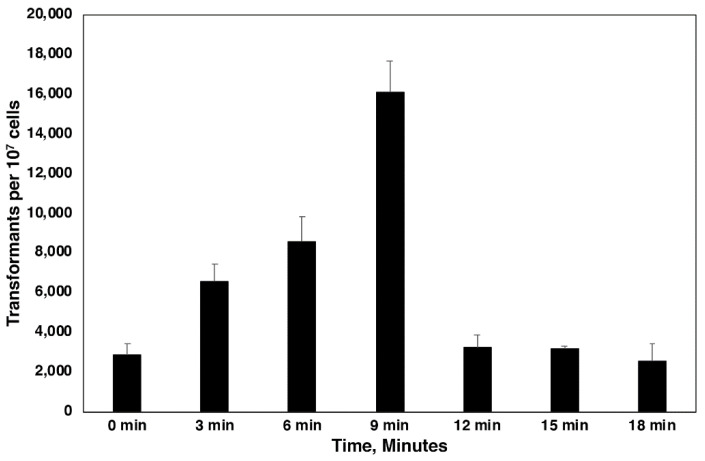
The effect of cell wall damage on transformation frequency. Yeast cells were vortexed with glass beads for the indicated times (X axis). Experiments were performed in triplicate, with three plates per experiment, and counted at 24 h. Colony counts are per 1 × 10^7^ *C. neoformans* cells. The 3, 6, and 9 time points were significantly different from each other and the 0, 12–18 time points, while the 12–18 time points were not significantly different from the 0 time point (*P* < 0.05).

Because most fungi have a rigid cell wall, we wanted to test the effect of cell wall damage on transformation efficiency in order to see if cell wall removal or damage would make cells more receptive to *A. tumefaciens* infection. Although it is possible to use spheroplasting in the removal of part or most of the cell wall in *C. neoformans*, it is an impractical transformation strategy. However, bead beating or vortexing with beads (method used in this experiment) is a quick and easy way to damage or remove fungal cell walls. It has also been shown to increase transformation frequencies of *A. tumefaciens* in other organisms [23]. Figure 2 shows the effect of increasing vortexing time on transformation frequency. Transformation frequency increased nearly linearly with 3 min vortexing intervals, up to an almost 8-fold increase over the no-vortexing control, until 9 min. Transformation frequency then rapidly decreased to control levels at the next time point (12 min) and beyond.

### 3.3. Acetosyringone Concentration

**Figure 3:**
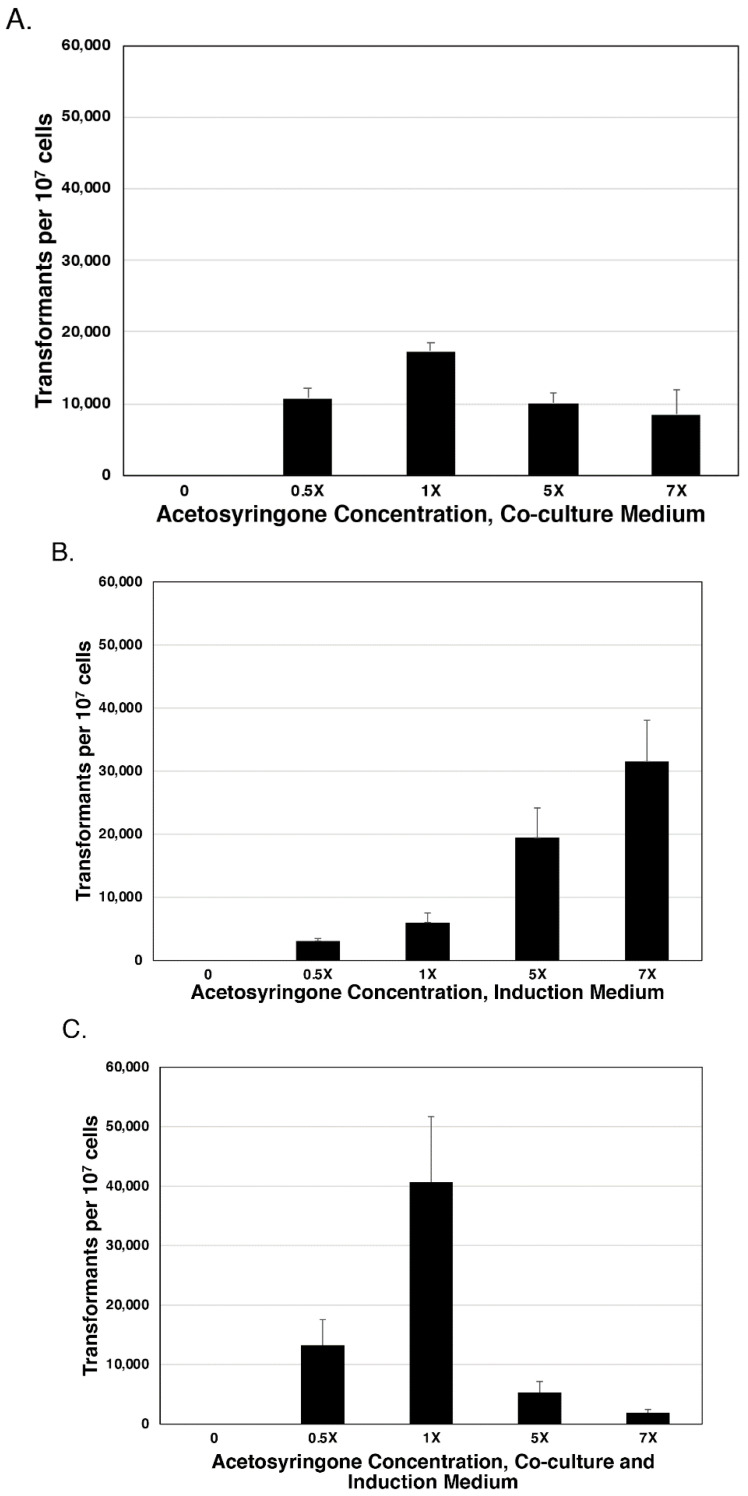
Acetosyringone effect on transformation efficiency. Acetosyringone was added at different times and different concentrations during the transformation. (**A**) Acetosyringone in co-culture medium only. An acetosyringone concentration of 1× was significantly different from 0.5×, 5×, and 7×, while there was no significant difference between 0.5×, 5×, and 7× at *P* < 0.05. (**B**) Acetosyringone in induction medium only. Transformation numbers increased steadily from 0 to 7×. Only 0.5× and 1× transformant numbers were not significantly different from each other at *P* < 0.05. (**C**) Acetosyringone in both the induction and co-culture media. Transformation frequency peaked at the 1× concentration of acetosyringone in both the induction and co-culture media, then dropped quickly at higher concentrations. The 1× concentration level was significantly different from all other concentrations at *P* < 0.05.

The effect of acetosyringone on transformation frequency was tested under three different conditions to provide an indication of when this compound exerts the greatest effect on transformation frequency. For each condition, if there was no acetosyringone in either medium, no transformants were recovered, demonstrating that *A. tumefaciens* needs acetosyringone in order to transfer the plasmid and that plasmid mobilization is likely to be tightly regulated in response to plant signals. When acetosyringone was added to co-culture agar alone, the optimum concentration was found to be the standard amount (200 μM) as more or less than the standard concentration, which reduced the transformant number from the highest level (~17,000/10^7^ cells)—observed at the standard concentration—to ~8500–10,000/10^7^ cells for the other concentrations (Figure 3A). When acetosyringone was included in the induction medium only, transformant levels increased steadily from 0 to ~31,000/10^7^ cells (Figure 3B). Only the 0.5× and 1× transformant numbers were not significantly different from each other at *P* < 0.05. The addition of acetosyringone to both the induction and co-culture media yielded the highest number of transformants for any condition, with the maximum number being ~41,000/10^7^ cells (Figure 3C). While the standard concentration level had the greatest effect on transformant number, the inclusion of acetosyringone in both the induction medium and co-culture medium resulted in more than twice as many transformants as acetosyringone in the induction medium only.

### 3.4. A. tumefaciens Transformation Is Enhanced with Increasing Agar Concentration

**Figure 4:**
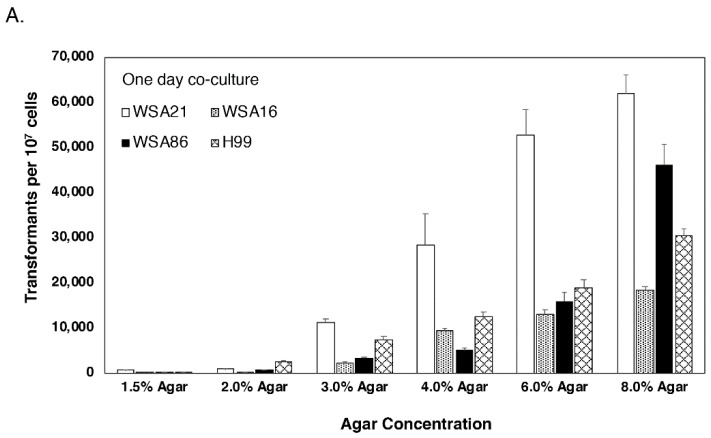

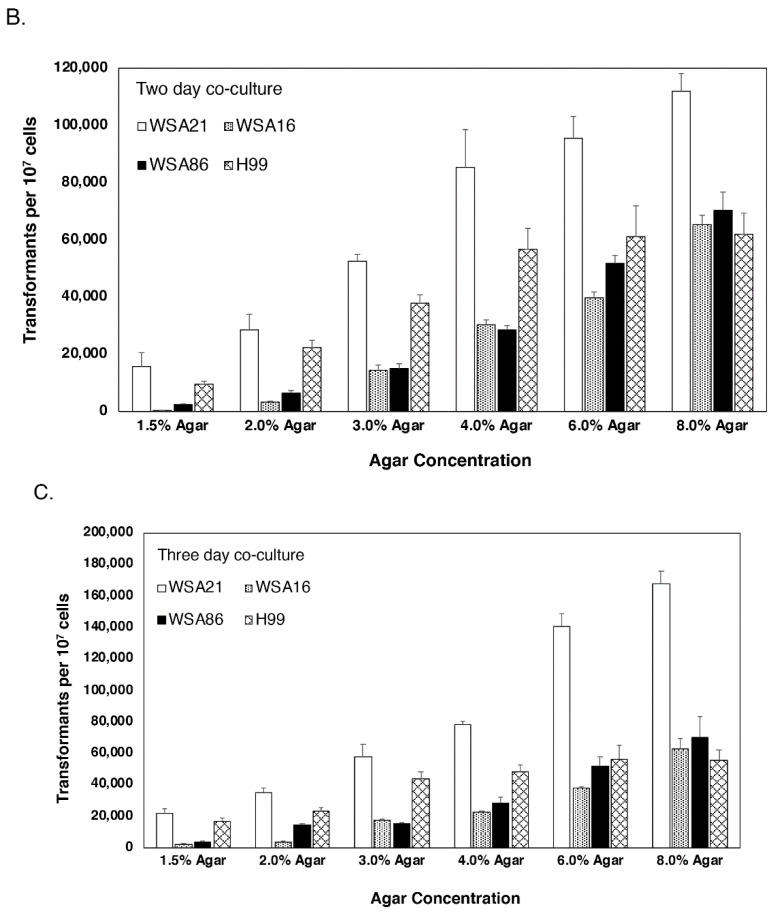
The effect of agar concentration on transformation frequency. For each of the four strains (WSA21, WSA16, WSA86, H99) increasing agar concentration resulted in an increase in transformation frequency. Transformant numbers increased each day, with WSA21 showing the highest number of transformants for each time point. Experiments were performed in triplicate with three plates per experiment. Colony counts are per 1 × 10^7^ *C. neoformans* cells. (**A**) 24 h incubation, (**B**) 48 h incubation, (**C**) 72 h incubation.

**Figure 5:**
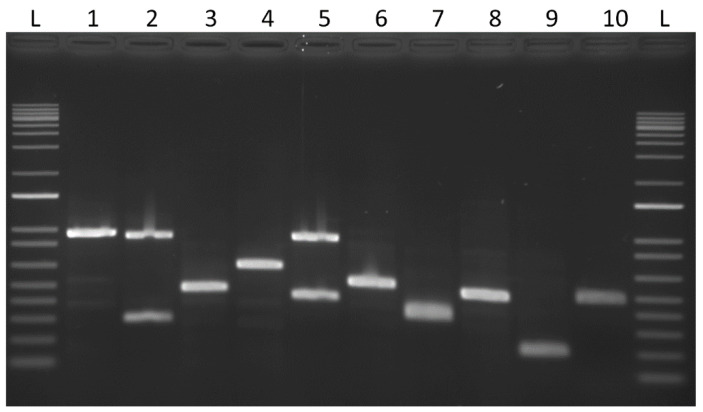
Vectorette PCR. An example of vectorette PCR to identify single insertions and multiple insertions. Lanes 2 and 5 show double insertions. For lanes that displayed same-sized bands, these PCR products were recovered and sequenced to rule out clonality.

Varying agar concentration can affect a number of different phenotypes in fungi. For example, *C. neoformans* is generally grown vegetatively on media with 2% agar, but when mating experiments are performed, 4% agar is used. Alternatively, the Spider medium [24] is often used to visualize hyphae in *Candida albicans* and is made with 1.5% agar instead of the more common 2% agar found in a nutrient medium such as YPD. Therefore, we tested a range of agar concentrations on transformation frequency through *A. tumefaciens*. Figure 4 shows that as agar concentration increased from the standard concentration of 1.5% to 8.0%, transformation efficiency increased with agar concentration for all four serotypes. In fact, we were only able to reach a concentration of 8% agar before the media became too unwieldy to work with, so it is unclear if we have reached the upper limit of agar density for co-culture medium or whether increasing agar concentration would continue to result in increased transformation frequencies. The fold increase from 1.5% to 8.0% varied with serotype. At a co-culture of one day on 8.0% agar, WSA21 (serotype D) yielded the most transformants, while WSA16 (serotype C) and WSA85 (serotype C) had the largest fold increase (more than 200×) compared to numbers on 1.5% agar (Figure 4A). At a co-culture period of two days, WSA16 again showed more than a 200-fold increase from 1.5% agar to 8.0% agar, while the remaining strains showed smaller increases of 7–29× (Figure 4B). For a three-day co-culture, the fold increase ranged from 3–28× total transformants per day (Figure 4C). In order to determine if the increase in transformation frequency led to an increase in multiple insertion frequency, insertion sites were screened by vectorette (Figure 5.) Multiple insertions appeared at a frequency of 25% on 1.5% agar, 31.81% on 4.0% agar, and 30.77% on 8% agar. There was a slight increase from 1.5% to 8% agar; however, differences were not significant (*P* < 0.05).

### 3.5. Infection Time Course

**Figure 6:**
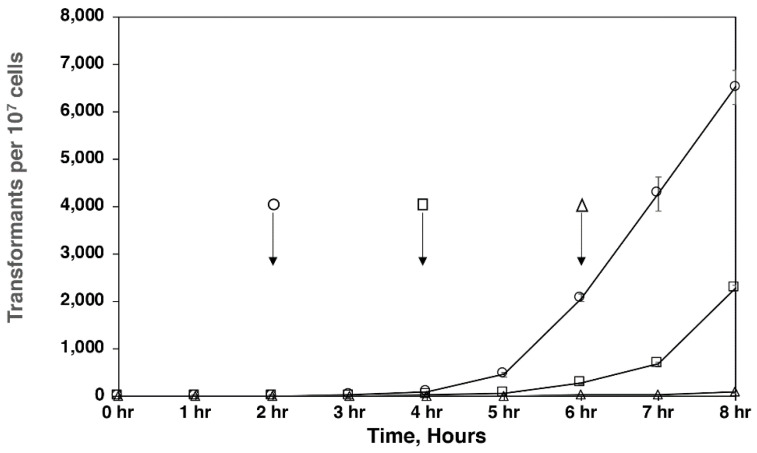
*A. tumefaciens* infection time course. *C. neoformans* and *A. tumefaciens* cells were mixed and filtered onto paper disks, which were then placed onto co-culture media with varying concentrations of agar for the indicated time. Experiments were performed in triplicate with three plates per experiment and counted hourly for 8 h. Colony counts are per 1 × 10^7^ *C. neoformans* cells. Open circles indicate cells plated onto co-culture media solidified with 8% agar. Open squares indicate cells plated onto co-culture media solidified with 4% agar. Open triangles indicate cells plated onto co-culture media solidified with 1.5% agar. Arrows indicate time at which the first colony appeared.

We were interested in determining when the Ti-plasmid transfer to yeast cells took place and designed the experiment based loosely on the classic work of Wollman et al. and their study of conjugation in *E. coli* [25]. In our case, we were screening for the appearance of G418 sulfate resistance, which is carried on the T-DNA that is transferred to the yeast cell from *A. tumefaciens* during the transformation process. Figure 6 shows that the first resistant colonies appeared at 2 h on 8% agar, which was 4 h earlier than colony appearance on 1.5% agar. Transformants continued to appear through the duration of the experiment with increasing frequency. Extending the length of the experiment to 24 h resulted in colonies continuously appearing, although as time proceeded, some of these transformants could have been due to the clonal replication of host yeast cells with the corresponding *A. tumefaciens* insert. More importantly, agar concentration affected the time of appearance of the first transformants, with increasing concentration resulting in earlier appearance and higher numbers of transformants.

## 4. Discussion

*Agrobacterium tumefaciens* has become increasingly popular as a molecular tool for manipulating fungi and has been used extensively for gene characterization in *C. neoformans* [7,19,26,27,28,29]. The utility of *A. tumefaciens* as a tool for studying *C. neoformans* is due to two main reasons. The first is that integrations tend to be once per cell, although multiple integrations can occur [1]. Second, integrations tend to occur randomly throughout the genome [1]. These characteristics make *A. tumefaciens* analogous to bacterial transposons, which can be used for insertional mutagenesis, although *A. tumefaciens* transformation occurs at much lower frequencies. Nonetheless, if a selectable phenotype is available, *A. tumefaciens* can be extremely valuable as a mutagenic tool because off-target damage, such as what would occur with chemical or UV mutagenesis, can be avoided. Furthermore, after selecting a transformant of interest, the location of the insertion site can quite easily be determined using a variety of methods [20]. A precise determination of insertion location within the genome is possible because genomes have been sequenced and annotated from multiple isolates representing the *C. neoformans* and *C. gattii* species complexes. In fact, this advantage is the case with any fungus that has an annotated genome. The ease of transformation, which consists mainly of growing yeast and bacterial strains to the appropriate stage and then collecting the mixture on a filter after making adjustments for CFU ratios, makes *A. tumefaciens* a potentially very powerful insertional mutagenesis tool.

The main drawback to the widespread use of *A. tumefaciens* in fungi is that in contrast to transposon-mediated insertional mutagenesis in bacteria, the *A. tumefaciens* transformation frequency in fungi is orders of magnitude lower than transposon insertional mutagenesis in bacteria. Fungal genomes are also larger than bacterial genomes and can contain various lengths of intergenic sequences that are non-coding. In fact, while numerous human fungal pathogens have been transformed by *A. tumefaciens*, the frequencies are too low to perform extensive mutagenesis screens. However, because of its widespread use in fungi, we were interested in investigating some of the variables that affect *A. tumefaciens* transformation frequency in an effort to determine if modifying one or more conditions could have a significant effect on transformation frequency. In this study, we looked at yeast to bacteria cell ratios, cell wall damage, acetosyringone concentration, and agar concentration. Some of these variables are commonly investigated during the development of new *A. tumefaciens* transformation systems, although the standard method that we used is very common among fungal *A. tumefaciens* transformation systems due to its robustness. Other variables that we did not investigate, which can affect transformation efficiency, include *A. tumefaciens* host strain [30], co-cultivation variables such as medium pH [31], temperature [32], and filter composition [33]. Additionally, while we investigated multiple serotypes (or species, depending on taxonomic preference), we only tested a single strain of each, which will not capture strain–strain variation, if it occurs.

Varying bacteria:yeast ratio did not identify any ratios that were better than the standard transformation ratio of 10:1 bacteria to yeast. Beginning with a 1:1 ratio, transformants increased until a ratio of 10:1 was reached, after which levels did not increase further, even at 50:1 bacteria to yeast. We did not test the effect of different ratios on multiple integration frequency; however, it is possible that at higher bacterial numbers, at some point, multiple plasmid transfers into the same cell could occur at high frequency, leading to less desirable multiple integrants. Therefore, while there is only a slight decrease in transformant numbers at ratios greater than 10:1, these levels should probably be avoided unless multiple insertion frequencies are tested due to the possible risk of increasing multiple integrations.

Although cell wall damage is probably too laborious to include in a protocol due to difficulties in standardization and the need to account for the reduction in cell viability because of damage, we saw significant differences in transformation frequencies as vortexing times increased up to a point, after which transformation frequency dropped off extensively. The later times that resulted in less transformants likely included a significant amount of cell death. Physical cell wall damage is probably cruder than light spheroplasting, albeit much easier; however, spheroplasting has been successfully used in fungal transformation by *A. tumefaciens* [34,35,36], again demonstrating that reduction in cell wall thickness or direct damage to the cell wall can affect transformation frequency. It is noteworthy that *C. neoformans* and *C. gattii* are encapsulated yeasts, yet the capsule does not appear to hinder *A. tumefaciens* transformation to any significant degree.

Acetosyringone in the co-culture medium did not improve transformation frequencies significantly above or below the standard concentration. However, a continuous increase in transformation frequency was seen when concentrations were increased in induction medium only. Interestingly, when acetosyringone was included in both media, the concentration that yielded the highest number of transformants was the amount used in the standard transformation protocol. Furthermore, increased concentrations resulted in a steady decrease in transformants, suggesting that too much acetosyringone can have an inhibitory effect on plasmid transfer, or possibly a toxic effect on the cells. It is worth noting that we only tested the effect of acetosyringone at a single exposure time. Xi et al. found increasing transformation frequencies with a longer exposure to acetosyringone [37]. Other variables combined with acetosyringone concentration could affect transformation frequency. For example, Manfroi et al. tested different acetosyringone concentrations in combination with pH and infection temperature and found significant differences depending on the combinations [38]. These studies suggest that there are other variables that could be investigated with regard to the acetosyringone effect on transformation; however, the relationship could be complex and laborious to investigate, but may be worth testing if other variables do not yield significant improvements.

While we found that the inclusion of acetosyringone in both the induction and co-culture media approximately doubled transformation frequency, we found agar concentration to have the most pronounced effect on transformation frequency. Depending on strain, agar concentration, and co-culture incubation time, we found increases in transformation frequency as high as 200-fold. We likely did not reach the upper limit since agar percentages higher than 8% were nearly impossible to work with. It is possible that alternate substrates, including solid surfaces, may yield even higher transformation frequencies, although access to moisture and nutrients could be a problem for non-agar-based surfaces. However, given that agar concentration had the most pronounced effect on transformation frequency, plating substrate may be the area to investigate in more detail to see if transformation frequency can be further increased, at least in *Cryptococcus* spp.

A surprising observation regarding the effect that agar concentration had on *A. tumefaciens* is the reduction of initial Ti-plasmid insertion time with increasing agar concentration. Colonies first began to appear at 2 h on 8% agar, in contrast to 1.5% agar where colonies first appeared at 6 h. The difference in infection time combined with the larger number of transformants that appear on more concentrated agar may suggest that the more concentrated agar leads to the more efficient attachment of bacteria to the yeast cell—perhaps both in the number of bacteria that successfully attach, and/or the success of the pore formation between the two cells. The firmer agar substrate may serve to enable a more stable pore channel scaffold; the agar concentration effect could also be simpler, perhaps because water is made less available on the agar surface, which could serve to destabilize bacteria/yeast cell contact and make pore formation more inefficient. The agar concentration variable had the most pronounced effect of all variables that we tested and should serve as a component of any *A. tumefaciens* transformation protocols.

In this study we investigated a number of variables that are used for *A. tumefaciens* transformation, and we specifically looked at existing and new parameters for *Cryptococcus* spp. Some variables did not improve transformation frequencies more than what has previously been reported, while others resulted in substantial improvements. Of the major human fungal pathogens, *A. tumefaciens* transformation of *C. neoformans* has yielded the most promising results. However, continued investigation of the *A. tumefaciens* system for this fungus may uncover additional improvements, and more importantly, serve as a foundation for developing new *A. tumefaciens* systems in other fungi, or for improving existing systems.

## Data Availability

Not applicable.
